# Colo-colonic intussusception secondary to Burkitt lymphoma with concurrent malignant small bowel mesh adhesion

**DOI:** 10.1093/omcr/omae095

**Published:** 2024-08-26

**Authors:** Mei Jing Ho, Faisal Syed, Wieslawa Mary Wielebinski, Kamal Galketiya

**Affiliations:** Department of Surgery, Nepean Hospital, Somerset Street, Kingswood, NSW 2747, Australia; Department of Surgery, Dubbo Base Hospital, Myall Street, Dubbo, NSW 2830, Australia; Histopathology Department, Dubbo Base Hospital, Myall Street, Dubbo, NSW 2830, Australia; Department of Surgery, Dubbo Base Hospital, Myall Street, Dubbo, NSW 2830, Australia

**Keywords:** Colo-colonic intussusception, Burkitt lymphoma, adult, mesh

## Abstract

Intussusception is a rare presentation in adult population and usually occurs secondary to an underlying pathology. We report an unusual case of a 28-year-old female who developed a colo-colonic intussusception secondary to Burkitt lymphoma which was managed with an extended right hemicolectomy. The case was further complicated by a segment of small bowel with malignant adhesion to a prosthetic mesh requiring resection of the involved segment of small bowel. We have discussed the significance of this case as well as general considerations in the surgical management of adult intussusception.

## Introduction

Intussusception is a relatively rare condition which typically affects paediatric populations, with 90% of cases presenting by 2 years of age [1]. In adults, it is often secondary to a pathological lead point like malignancy [2, 3]. Extra-nodal type Burkitt lymphoma is a rapidly growing B-cell malignant neoplasm typically characterised by chromosome 8 translation involving the c-MYC oncogene, with common sites of extra-nodal manifestation including the ileum and caecum [1]. Whilst Burkitt lymphoma is the most common cause of intussusception of children over the age of 4, it is an uncommon cause of intussusception in adults [4].

## Case report

A 28-year-old Caucasian female presented to a rural ED with right upper quadrant abdominal pain 5 days after a 13-week miscarriage and subsequent dilation, curettage and removal of retained products of conception. There was no report of obstructive symptoms with bowels opening that day and no history of nausea or vomiting. Her medical history was significant for asthma, migraines, reflux, anxiety and depression. In terms of surgical history, she had a laparoscopic appendicectomy 18 months prior to presentation. A week later, she presented with gallstone pancreatitis with a distal common bile duct stone identified on Magnetic retrograde cholangiopancreatography (MRCP). She underwent an Endoscopic retrograde cholangiopancreatography (ERCP) followed by an elective laparoscopic cholecystectomy a month later. Despite these procedures and a normal intra-operative cholangiogram, she had recurrent episodes of cholangitis requiring multiple ERCPs and biliary stenting for a benign CBD stricture with her last ERCP was 3 months prior to this presentation. Furthermore 5 months prior, she presented with a painful but reducible umbilical incisional hernia containing small bowel which was laparoscopically repaired with a 12 cm Symbotex mesh secured with suture and AbsorbaTacks.

**Figure 1:**
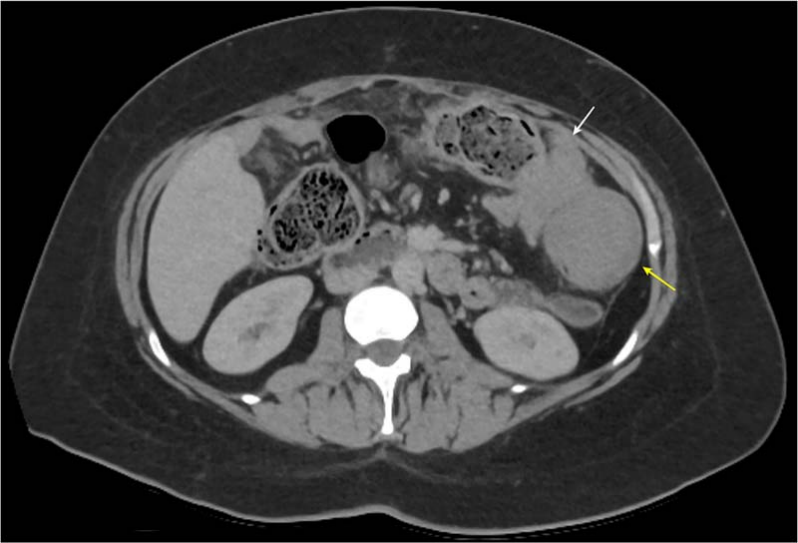
Abdominal CT axial slice demonstrating colo-colonic intussusception at the splenic flexure and a colonic soft tissue mass just distal.

On presentation, she was mildly tachycardic with a normal blood pressure. Blood results were all within normal limits. A computed tomography (CT) of her abdomen and pelvis revealed colo-colonic intussusception with a soft tissue mass in proximity to the splenic flexure (Fig. 1). A review of her last imaging performed 3 months ago showed no evidence of a colonic soft tissue mass in that location. She also had a colonoscopy 1 year ago for investigation of diarrhoea, which showed increased mucosal eosinophils of the terminal ileum and active chronic inflammation of the colon with eosinophilia.

**Figure 2:**
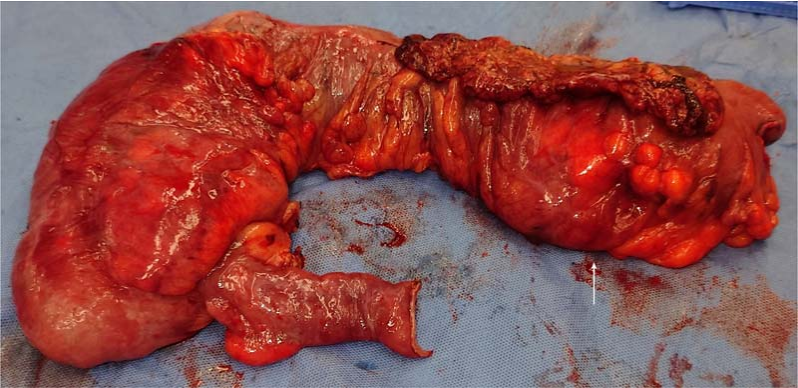
Clinical photo of the operative specimen with a large colonic mass (white arrow).

The patient was resuscitated with intravenous fluids and consented for an urgent laparotomy. Intra-operatively, a large polypoid colonic mass of the mid-transverse colon (Fig. 2) with associated lymphadenopathy were observed. Furthermore, a segment of the proximal ileum was densely adherent to the mesh from her previous incisional hernia. There were no liver lesions or evidence of obvious peritoneal disease. As such, an extended right hemicolectomy and anastomosis was performed. As the adherent small bowel was unable to be safely dissected off the mesh, a small bowel resection and anastomosis was also performed.

**Figure 3:**
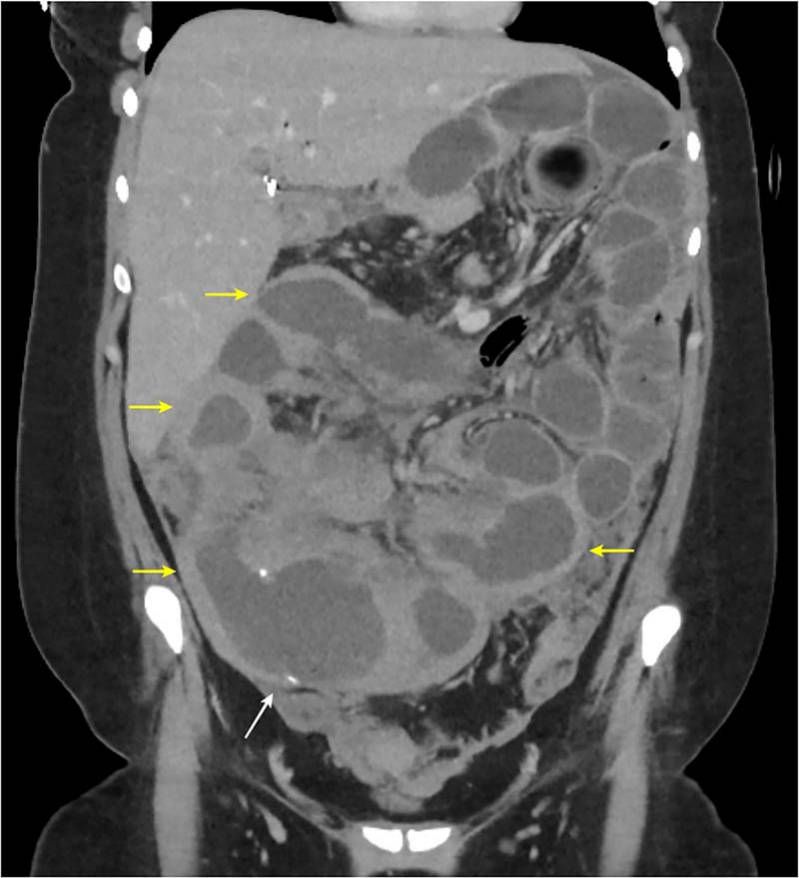
Abdominal CT coronal slice demonstrating multiple loops of dilated fluid filled small bowel with significant wall thickening.

The patient made an uneventful recovery and was discharged home 5 days later. However, she re-presented 11 days later with right-sided abdominal pain, abdominal distension and vomiting. A repeat CT demonstrated thickened and dilated loops of small bowel and a collapsed colon, consistent with a high-grade small bowel obstruction with no evidence of anastomotic complications (Fig. 3). By this stage, her histopathology had returned with a diagnosis of high-grade Burkitt lymphoma extensively involving the large bowel, resection margins, 53 pericolic lymph nodes and omentum, as well as serosal involvement of the segment of resected small bowel. Histopathological staining indicated a Ki-67 proliferation index of almost 100% and diffuse positive staining for c-MYC.

She was managed non-operatively with nasogastric decompression, bowel rest and intravenous hydration. The Haematology team was consulted and commenced her on steroids. As there was no improvement in her obstructive symptoms, thought to be secondary to her extensive lymphoma, she was urgently transferred to a major tertiary centre for ongoing treatment and commencement of chemotherapy.

## Discussion

Intussusception occurs when a segment of bowel telescopes into an adjacent bowel segment. While it can occur at any age, it is most common in children. In contrast, it is a relatively rare clinical entity in adults, accounting for only 5% of cases and 1-5% of intestinal obstruction [5–9]. In paediatric populations, intussusception is typically idiopathic with a small number being secondary to a lead point such as a Meckel’s diverticulum, a benign or malignant lesion. This aetiology is reversed in adults, with majority of cases secondary to a pathological or neoplastic process [1, 5–8]. The exact mechanism on intussusception remains unknown. However, it is postulated that the presence of a bowel lesion changes the normal peristaltic activity, which serves as the lead point to trigger the invagination [5, 9]. Whilst there are some case reports of intussusception secondary to Burkitt lymphoma in paediatric and adult populations, it appears that colo-colonic intussusception is a less common phenomenon in both demographics [1, 8]. There is a case of ileocolic intussusception secondary to Burkitt lymphoma in a 17-year-old male with nodal involvement [10]. In addition, Sendra-Fernandez et. al described another case of Burkitt lymphoma with extranodal involvement [11]. Our current report adds to the paucity of literature primary colorectal lymphoma as a cause of adult intussusception.

The clinical presentation in adult intussusception is often chronic and non-specific in nature. Abdominal pain is the most common presenting complaint. Other symptoms include nausea, vomiting, altered bowel habits and haematochezia [5, 7, 9]. There are multiple imaging modalities used in the diagnosis of intussusception, namely, plain abdominal X-ray, abdominal ultrasound, barium studies and CT abdominal scans. Nevertheless, the diagnosis of intussusception is often made using abdominal CT imaging, given its diagnostic yield of approximately 58%–100% [9] and facilitates the identification of the underlying aetiology. The CT feature includes a ‘target sign’ which can usually be observed on the sagittal view [5, 7, 9].

In adults, the definitive approach to intussusception is surgery, especially once a malignant lesion has been identified as a potential lead point [5, 7, 8]. This usually involves an oncological resection and primary anastomosis of the involved segment of bowel. Whilst endoscopic reduction can be attempted if no such lesion has been identified, ultimately surgical resection may be required if there is any endoscopic or clinical evidence of perforation, enteric or colonic ischemia, non-resolving obstruction or a mucosal lesion [6]. Given the patient had an uneventful colonoscopy a year prior and that there was an obvious soft tissue colonic lesion on imaging, our patient was treated surgically with a resection and primary anastomosis of the involved segment of bowel.
